# Histamine induced high mobility group box-1 release from vascular endothelial cells through H_1_ receptor

**DOI:** 10.3389/fimmu.2022.930683

**Published:** 2022-10-05

**Authors:** Shangze Gao, Keyue Liu, Wenhan Ku, Dengli Wang, Hidenori Wake, Handong Qiao, Kiyoshi Teshigawara, Masahiro Nishibori

**Affiliations:** ^1^ Department of Pharmacology, Okayama University Graduate School of Medicine, Dentistry and Pharmaceutical Sciences, Okayama, Japan; ^2^ School of Pharmaceutical Sciences, Tsinghua University, Beijing, China; ^3^ Tsinghua-Peking Center for Life Sciences, Tsinghua University, Beijing, China; ^4^ Department of Translational Research and Drug Development, Okayama University Graduate School of Medicine, Dentistry and Pharmaceutical Sciences, Okayama, Japan

**Keywords:** Histamine, HMGB1, vascular endothelial cell, H_1_ receptor, hypotension

## Abstract

**Background:**

Systemic allergic reaction is characterized by vasodilation and vascular leakage, which causes a rapid, precipitous and sustained decrease in arterial blood pressure with a concomitant decrease of cardiac output. Histamine is a major mediator released by mast cells in allergic inflammation and response. It causes a cascade of inflammation and strongly increases vascular permeability within minutes through its four G-protein-coupled receptors (GPCRs) on endothelial cells. High mobility group box-1 (HMGB1), a nonhistone chromatin-binding nuclear protein, can be actively secreted into the extracellular space by endothelial cells. HMGB1 has been reported to exert pro-inflammatory effects on endothelial cells and to increase vascular endothelial permeability. However, the relationship between histamine and HMGB1-mediated signaling in vascular endothelial cells and the role of HMGB1 in anaphylactic-induced hypotension have never been studied.

**Methods and results:**

EA.hy 926 cells were treated with different concentrations of histamine for the indicated periods. The results showed that histamine induced HMGB1 translocation and release from the endothelial cells in a concentration- and time-dependent manner. These effects of histamine were concentration-dependently inhibited by *d*-chlorpheniramine, a specific H_1_ receptor antagonist, but not by H_2_ or H_3/4_ receptor antagonists. Moreover, an H_1_-specific agonist, 2-pyridylethylamine, mimicked the effects of histamine, whereas an H_2_-receptor agonist, 4-methylhistamine, did not. Adrenaline and noradrenaline, which are commonly used in the clinical treatment of anaphylactic shock, also inhibited the histamine-induced HMGB1 translocation in endothelial cells. We therefore established a rat model of allergic shock by i.v. injection of compound 48/80, a potent histamine-releasing agent. The plasma HMGB1 levels in compound 48/80-injected rats were higher than those in controls. Moreover, the treatment with anti-HMGB1 antibody successfully facilitated the recovery from compound 48/80-induced hypotension.

**Conclusion:**

Histamine induces HMGB1 release from vascular endothelial cells solely through H_1_ receptor stimulation. Anti-HMGB1 therapy may provide a novel treatment for life-threatening systemic anaphylaxis.

## Introduction

Histamine, 2-(4-imodazole)-ethylamine, is synthesized from L-histidine exclusively by histidine decarboxylase and can be produced by various cells, including central nervous system neurons, vascular endothelial cells (VECs), gastric mucosa parietal cells, mast cells, basophils and lymphocytes (1). Histamine plays an important role both in normal human physiology as well as in various pathologies, such as allergic inflammation and response (2–4), gastric acid secretion (5), neurotransmission in the central nervous system (6, 7), and the regulation of innate immune response (8, 9). Anaphylactic shock (AS) often results from an immunoglobulin E (IgE)-mediated systemic allergic reaction. AS is characterized by vasodilation and vascular leakage, and causes a rapid decrease in systemic arterial blood pressure that contributes to the onset of hypotension (10–12). Histamine is a major inducer of vascular hyperpermeability, and thus it is a central component of permeability-related human pathologies, such as allergy and anaphylaxis (13). Histamine released from mast cells and basophils triggers acute symptoms due to its very rapid activity on the vascular endothelium and bronchial and smooth muscle cells, which leads to a rapid increase in vascular permeability within minutes. Histamine-induced production of NO through eNOS in the VECs also results in NO diffusion into the smooth muscle cell layer in the vessel wall and dilates smooth muscle cells by activating cytosolic guanylate cyclase (14).

Histamine acts through its four G-protein-coupled receptors (GPCRs), histamine receptors 1 to 4 (H_1_R to H_4_R) (15, 16). These vascular effects of histamine are in general mediated by histamine H_1_-receptor and constitute the main actions of histamine on blood vessels (14), whereas the H_2_-receptor modifies gastric acid secretion, airway mucus production, and vascular permeability (16). The H_3_-receptor has been shown to be involved in neuron-inflammatory diseases (17). The H_4_-receptor plays an important role in allergy and inflammation (18).

High mobility group box-1 (HMGB1) is a ubiquitous nuclear protein that binds to chromatin DNA, thereby regulating transcription activity and maintaining chromatin structure (19, 20). Under injurious stimuli and stress, HMGB1 is translocated from nuclei to the extracellular space through the cytosolic compartment (21). Extracellular HMGB1 is now recognized as a representative damage-associated molecular pattern (DAMP) and has been shown to be involved in many diseases as an inflammation enhancer through the direct stimulation of TLR-4/2 and RAGE as well as through complex formation with IL-1β and CXCL12, with subsequent enhancement of the activation of cognate receptors (22). In addition, HMGB1 may carry LPS to Kupffer cells, leading to the efficient production of inflammatory cytokines in a gasdermin- and caspase-dependent manner (23). Among the diverse range of effects of HMGB1 on cellular responses, the effects on capillary blood vessels are especially notable. In an ischemic/reperfusion model in rats, it was demonstrated that HMGB1 released from neurons and other cells directly affected the BBB-constituting cells, VECs and pericytes, leading to increased permeability and brain edema formation (24, 25). In peripheral capillary endothelial cells in culture, an HMGB1-induced contractile response and subsequent increase in permeability were observed (26). However, whether histamine-induced vascular permeability is related with HMGB1 release from endothelial cells has never been investigated.

VECs should be controlled precisely depending on the micromilieu. Under a resting condition, the luminal surface of VECs is maintained in an anti-coagulation state. At the same time, the interaction between endothelial cells and blood cells is expected to be kept minimal. However, once the disruption of vascular walls occurs or agonistic stimuli reach the endothelial cells, rapid changes in the cellular phenotype should occur, including phenotypic changes related to the direction of coagulation or facilitation of inflammation through the migration of infiltrating leukocytes. To elucidate these phenomena, numerous bioactive factors on VECs that finely tune the state of VECs have been identified. In the previous study (27), we demonstrated that LPS and TNF-α induced the release of HMGB1 from VECs in culture, associated with the production of the inflammatory cytokines and the expression of adhesion molecules on their surface although the signaling pathways leading to the translocation of HMGB1 remain unclear.

In the present study, we found that a classical mediator of inflammation, histamine, concentration-dependently caused HMGB1 release from VECs in culture through the stimulation of specific H_1_-receptor. Moreover, our findings suggest that the hypotensive response induced *in vivo* by a liberator of histamine from mast cells may be mediated in part by HMGB1. These findings will provide new insights into our understanding of vascular biology and could lead to therapeutic strategies for histamine-induced vascular reactions in allergy and anaphylaxis.

## Materials and methods

### Chemicals and reagents

Histamine dihydrochloride was obtained from Nakalai Tesque (Kyoto, Japan). 2-Pyridylethylamine dihydrochloride and 4-methylhistamine dihydrochloride were gifts from Drs. W.A.M. Duncan and G.J. Durant (The Research Institute, Smith Kline & French Laboratories, Welwyn Garden City, Herts). d-Chlorpheniramine maleate and famotidine were obtained from Takeda Pharmaceutical Company (Osaka, Japan) and Yamanouchi Pharmaceuticals (Tokyo), respectively. Compound 48/80 trihydrochloride was obtained from Funakoshi (Tokyo).

### Cell cultures

EA.hy 926 endothelial cells (ATCC Cat# CRL-2922, RRID:CVCL_3901), a hybridoma of human umbilical vein endothelial cells (HUVECs) and the human epithelial cell line A549, were cultured using Dulbecco’s modified Eagle medium (DMEM, #D6546, Sigma, St. Louis, MO) supplemented with 10% fetal bovine serum (Gibco, Grand Island, NY), 5% L-glutamine (#G7513, Sigma), and 10% penicillin/streptomycin (Gibco) in 5% CO_2_ at 37°C. After reaching confluence, the EA.hy 926 cells were detached from culture flasks using 0.25% Trypsin-EDTA (Gibco), washed, and resuspended in DMEM. The cells were used between the third and sixth passage in our experiments.

### Immunostaining assay

EA.hy 926 cells were pretreated with FBS-free medium for 1 h before being stimulated with different concentrations of histamine (Nakalai Tesque) for the indicated periods. The cells were then fixed with 4% paraformaldehyde (Wako Pure Chemical Industry, Osaka, Japan) and blocked with 3% bovine serum albumin (BSA), after which the cells were stained by anti-HMGB1 Ab (rabbit, Sigma, RRID:AB_444360) for 1 h at 37°C followed by Alexa Fluor 488-labeled anti-rabbit/mouse IgG. Cell nuclei were stained with DAPI for 5 min, and then observed using a confocal microscope (LSM 780, Carl Zeiss).

### Cell viability

EA.hy 926 cells were plated in 96-well plates at 5×10^5^ overnight, and then pre-incubated with histamine at the indicated concentrations. The cells were then incubated with MTT at 37 °C for 4 h by adding 10 μl of 5 ng/ml MTT solution to each well. After removal of the cell supernatant, 200 μl of DMSO was added to each well to dissolve the crystals. The absorbance of each well was measured using a microplate reader (model 680; Bio-Rad) at 570 nm wavelength, and the optical density (OD) value was recorded.

### Quantitative real-time polymerase chain reaction (qRT-PCR)

EA.hy 926 endothelial cells were harvested and mRNA was extracted using an RNeasy mini kit (Qiagen). Total RNA (1 μg/sample) was incubated with the components of the PrimeScriptR RT reagent kit (Takara Bio, Shiga, Japan; Code No. RR036A) at 37°C for 15 min. The cDNA was then amplified with a SYBRR Premix Ex Taq™ (Tli RNaseH Plus) Kit (Takara Bio, Shiga, Japan; Code No. RR420A) with a Light Cycler (Roche, Basel, Switzerland). All operations followed the manufacturer’s protocol. The mRNA expressions of all genes were normalized to the housekeeping gene, β-actin. The fold changes between groups were calculated using the Ct value with the 2^−ΔΔCt^ method (ΔCt = Ct _target gene_ – Ct _β−actin_). Primers were designed according to published sequences (see the Supplementary materials and methods; **Table S1**).

### Enzyme-linked immunosorbent assay (ELISA)

To determine HMGB1 levels in plasma, blood samples were collected through the rat heart under deep anesthesia, then centrifuged for 10 min at 3000 rpm. The cell culture medium was collected after treatment to measure the release of HMGB1 from the cell to the supernatant, and then centrifuged for 10 min at 3000 rpm. HMGB1 was detected by using an ELISA kit (Shino-Test Co., Sagamihara, Japan) according to the manufacturer’s instructions.

### Effects of histamine receptor subtype-selective agonists and antagonists on HMGB1 mobilization

EA.hy 926 cells were prepared as described above. To determine the effects of receptor subtype-selective antagonists on histamine-induced translocation of HMGB1, EA.hy 926 cells were preincubated with 1 μM *d*-chlorpheniramine (H_1_-selective antagonist), famotidine (H_2_-selective antagonist) or thioperamide (H_3_/H_4_-selective antagonist) for 1 h. The cells were then stimulated with histamine (1 μM) for 12 h. To determine the effects of receptor subtype-selective agonists, 2-pyridylethylamine (H_1_-selective agonist) or 4-methylhistamine (H_2_-selective agonist) was used instead of histamine. Immunostaining of HMGB1 was performed as described above.

### Animals

Experiments were performed using 8-week-old male Wistar rats (body weight: 250 ± 15 g) housed in groups of three in polypropylene cages with a 12-h light-dark cycle at 24–26°C and ad libitum food and water. After a 1-week acclimatization, rats were divided among three groups of six rats each: an experimental group consisting of sensitized rats treated with PBS 1 min after shock induction; an anti-HMGB1 mAb group consisting of sensitized rats treated with α-HMGB1 mAb (2 mg/kg) 1 min after shock induction; and an anti-KLH mAb group consisting of sensitized rats treated with α-KLH mAb (2 mg/kg) 1 min after shock induction.

### Anaphylactic shock animal model

Rats were anesthetized with pentobarbital sodium solution (40 mg/kg) administered intraperitoneally. Then, the tissue was bluntly separated, the white ligament of the left leg was found, and the femoral artery was exposed by clamping the hemostatic forceps. Approximately 2 cm of the femoral artery was isolated, a NO. 4-0 surgical suture was passed through the radial and distal ends, and ligation was performed at the distal ends. Arterial puncture was performed with a 24G trocar between the two wires, and then the needle was removed. A blood pressure measuring device was connected to the end of the trocar, and the trocar was fixed to a real-time blood pressure recording system (Shino Test Co.) *via* a pressure transducer to measure the systolic, diastolic, and mean arterial blood pressure (MAP) and heart rate (HR). To prepare the anaphylaxis model, rats were administered a mast cell degranulation agent, compound 48/80, at a dose of 0.5 mg/kg body weight through the tail vein. After AS induction, rats in the three groups were administered with PBS, anti-KLH antibody (2 mg/kg) or anti-HMGB1 antibody (2 mg/kg) through the tail vein, respectively. Measurement of hemodynamic parameters was performed every 5 min for a period of 30 min before the compound 48/80 challenge. The hemodynamic parameters were recorded for 60 min at 1 min intervals after the compound 48/80 challenge.

### Statistical analysis

The data were analyzed with GraphPad Prism software ver. 6.01 (GraphPad, San Diego, CA). All values are presented as the means ± SEM and were analyzed by an analysis of variance (ANOVA) followed by Bonferroni’s test or *post hoc* Fisher test when the F statistic was significant. Probability (p) values <0.05 were considered significant. At least three independent experiments were performed for all of the assays.

## Results

### Histamine induced HMGB1 translocation and release from VECs

**Figure 1:**
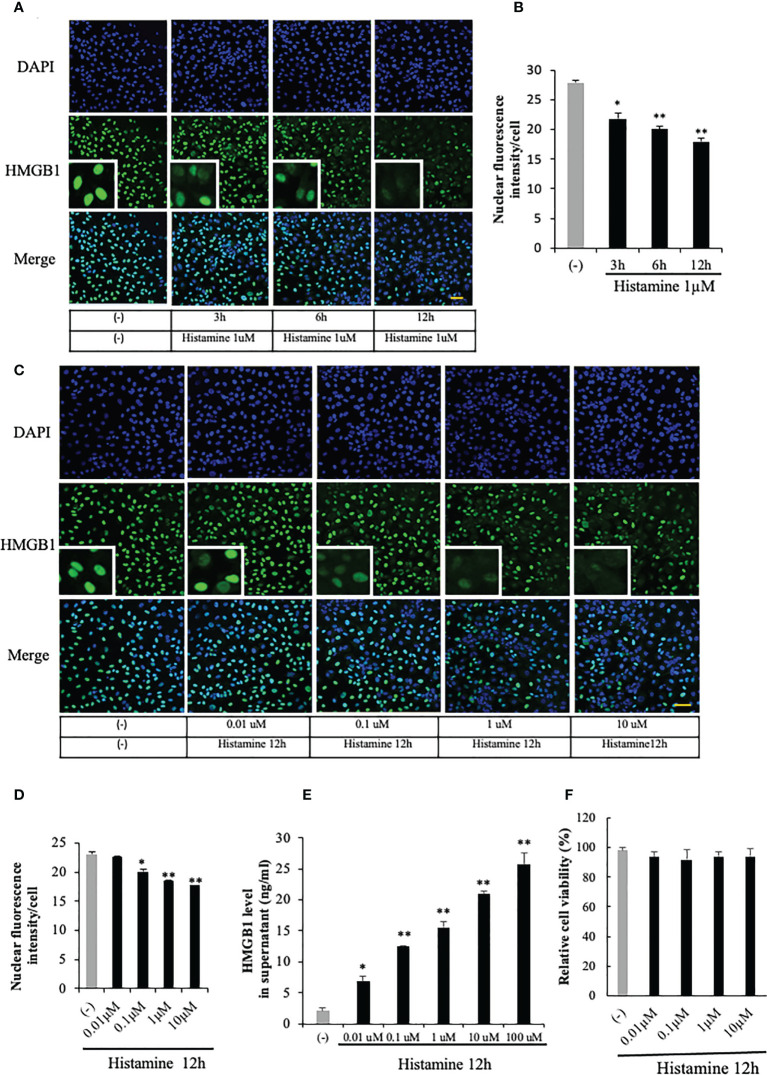
HMGB1 translocation and release from VECs after histamine stimulation. **(A)** EA.hy 926 endothelial cells were stimulated with histamine (1 μM) for the periods indicated. HMGB1 was observed by immunostaining with green fluorescence and cell nucleus was observed by blue fluorescence after staining with DAPI. Scale bar = 10 μm, (n=5 per group). **(B)** The translocation of HMGB1 was quantified by the residual presence of the HMGB1 (green fluorescence) in the cell nucleus in each cell. The results were quantified by ImageJ software and are expressed as the ratio of total nuclear HMGB1 intensity/cell numbers. **(C**, **D)** EA.hy 926 cells were stimulated with the indicated concentrations of histamine for 12 h, and the HMGB1 in the nucleus was determined by the immunostaining (n=5 per group). **(E)** Endothelial cells were cultured for 12 h with different concentrations of histamine. The cell culture medium was collected and analyzed for HMGB1 release by ELISA. All results are the means ± SEM of five different experiments. **(F)** Effects of histamine on the viability of EA.hy 926 cells. EA.hy 926 cells were stimulated with different concentrations of histamine for 12 h. The cells were then incubated with MTT at 37°C for 4 h by adding 10 μl of 5 ng/ml MTT solution to each well. After removal of the cell supernatant, 200 μl of DMSO was added to each well to dissolve the crystals. The OD value was recorded using a microplate reader at 570 nm wavelength. All results are the means ± SEM of three different experiments, (n=5 per group). Statistical analyses were conducted by one-way ANOVA followed by the *post hoc* Fisher test. ^*^p<0.05, ^**^p<0.01 vs. control in the absence of histamine.

HMGB1 was exclusively localized in the nuclear compartment in the EA.hy 926 VECs under a resting condition (**Figure 1A**). Histamine (1 μM) time-dependently induced the translocation of HMGB1 from the nuclei to cytosolic compartment. The translocation of HMGB1 was quantified by the fluorescence intensity of HMGB1 remaining in the cell nuclei of endothelial cells after histamine stimulation (**Figure 1B**). It appeared that the immunoreactivity of HMGB1 in the nuclei was time-dependently decreased whereas that in the cytosolic compartment was increased (Figure1A). The effects of histamine on HMGB1 translocation at 12 h were concentration-dependent at concentrations from 0.01 μM to 10 μM (**Figures 1C, D**). To determine whether HMGB1 was further released into the cell culture media, we determined the HMGB1 levels in the supernatant with ELISA. As shown in **Figure 1E**, HMGB1 was released from VECs into the media after stimulation with histamine in a concentration-dependent manner.

HMGB1 can be actively released from cells in response to various stimuli and also passively released from cells during cell necrosis or apoptosis (27). In order to clarify whether HMGB1 was actively or passively released from the VECs after the stimulation with histamine, we evaluated the cell viability after the histamine stimulation (**Figure 1F**). The results showed that the stimulation with different concentrations of histamine did not change the cell viability of VECs (**Figure 1F**), which means that histamine actively induced the translocation and release of HMGB1 from VECs.

### Involvement of H_1_ receptor in the effects of histamine on HMGB1 translocation and release from VECs

**Figure 2:**
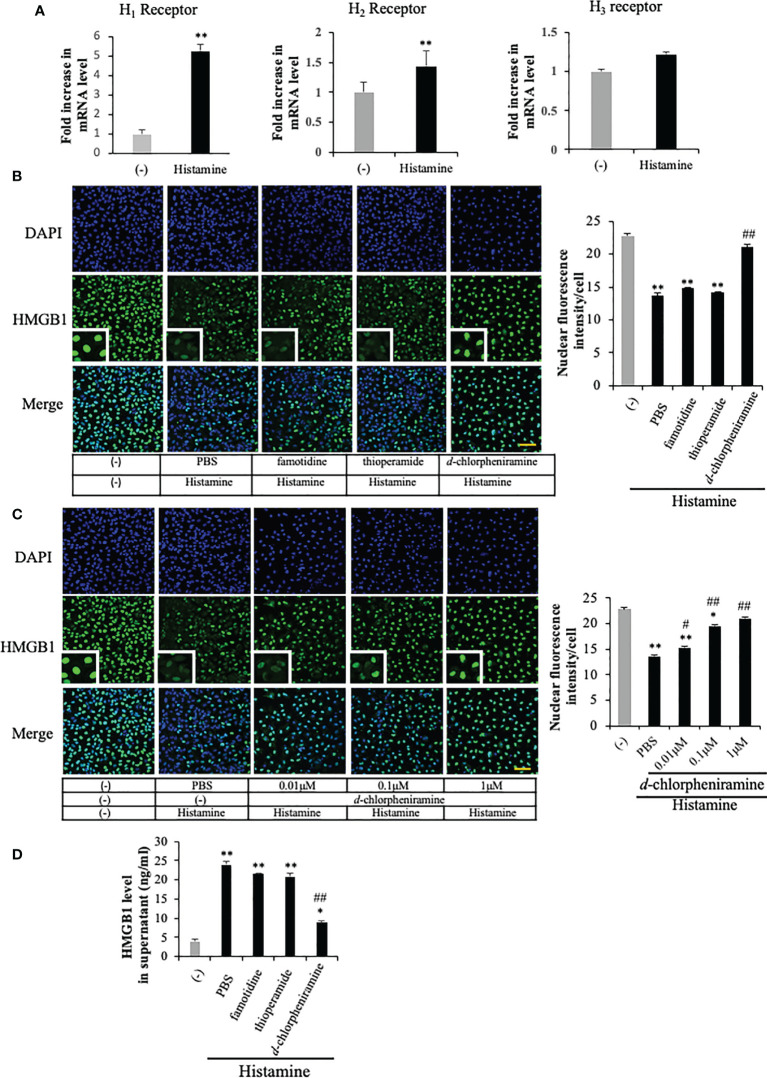
The involvement of histamine receptor subtypes in the histamine-induced HMGB1 release in VECs. **(A)** EA.hy 926 cells were cultured with histamine (1 μM) for 12 h. The mRNA expression of each histamine receptor in the presence or absence of histamine in the cells was measured by quantitative RT-PCR. The results were normalized to the expression of β-actin and are expressed as the mean ± SEM (n=5 per group). **(B)** EA.hy 926 cells were preincubated with each antagonist for 1 h before stimulation with histamine (1 μM). HMGB1 translocation was determined by immunostaining at 12 h after histamine stimulation. Scale bar = 10 μm. **(C)** Different concentrations of *d*-chloropheniramine were preincubated with the EA.hy 926 cells for 1 h before stimulation with histamine (1 μM) for 12 h. HMGB1 in the cell nucleus is quantified in the right panel of each group as the means ± SEM (n=5 per group). **(D)** EA.hy 926 cells were preincubated with each antagonist (1 μM) for 1 h. At 12 h after stimulation with histamine (1 μM), the amount of HMGB1 released into the medium was determined. Statistical analyses were conducted by one-way ANOVA followed by the *post hoc* Fisher test. ^*^p<0.05, ^**^p< 0.01 vs. control in the absence of histamine, ^#^p<0.05, ^##^p<0.01 vs. histamine-PBS group.

**Figure 3:**
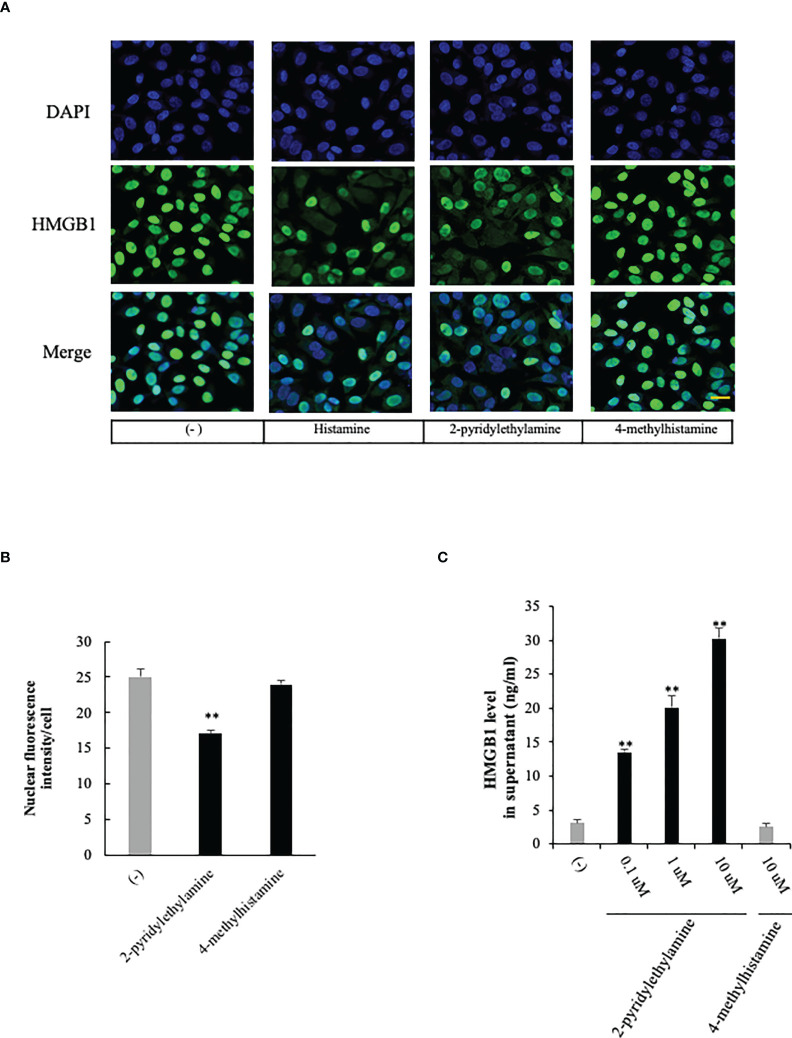
Effects of selective histamine receptor agonists on HMGB1 release from VECs. **(A)** EA.hy 926 cells were incubated with 2-pyridylethylamine (H_1_R-selective agonist) or 4 methylhitamine (H_2_R-selective agonist) for 6 h. HMGB1 translocation was observed with immunostaining as described in **Figure 1**. Scale bar = 10 μm. **(B)** The results were quantified by ImageJ software and are expressed as the ratio of total nuclear HMGB1 intensity/cell numbers. **(C)** The cell culture medium was collected and the HMGB1 released into media was measured by ELISA. All results are the means ± SEM of five different experiments, (n=5 per group). One-way ANOVA followed by the *post hoc* Fisher test. **p<0.01 vs. control in the absence of agonist.

Histamine acts through its four G-protein-coupled receptors (GPCRs), histamine receptors 1 to 4 (H_1_R to H_4_R) (9). To examine the effects of histamine on HMGB1 translocation and release from VECs, we first used RT-PCR to confirm the expression of histamine receptor subtypes H_1_, H_2_, and H_3_ in EA.hy 926 cells (**Figure 2A**). The expressions of H_1_R and H_2_R mRNA were increased 5-fold and 45%, respectively, after the incubation with histamine (1 μM) for 12 h, whereas that of H_3_R mRNA was not changed (**Figure 2A**). To examine which receptor was responsible for the histamine-induced translocation and release of HMGB1 from VECs, the receptor subtype-specific antagonists, *d*-chloropheniramine for H_1_R, famotidine for H_2_R and thioperamide for H_3/4_R, were used. The cells were preincubated with one of the antagonists for 1 h before stimulation with histamine (1 μM). The translocation and release of HMGB1 were evaluated 12 h thereafter (**Figures 2B–D**). An H_1_R-selective antagonist, *d*-chloropheniramine (1 μM), but not either famotidine (1 μM) or thioperamide (1 μM), inhibited the translocation induced by histamine (1 μM) (**Figure 2B**). The inhibitory effects of *d*-chloropheniramine were concentration-dependent (0.01–1 μM) (**Figure 2C**). We also confirmed that the secretion of HMGB1 into media induced by histamine (1 μM) was antagonized solely by *d*-chloropheniramine (1 μM). Moreover, an H_1_R-selective agonist, 2-pyridylethylamine (28), but not an H_2_R-selective agonist, 4-methylhitamine (29), mimicked the effects of histamine in regard to HMGB1 translocation (**Figures 3A, B**) and release into media (**Figure 3C**). These results as a whole indicated that the receptor subtypes involved in histamine-induced translocation and release of HMGB1 in VECs was H_1_ receptor.

### Calcium-dependency of the effects of histamine on HMGB1 translocation and release from VECs

**Figure 4:**
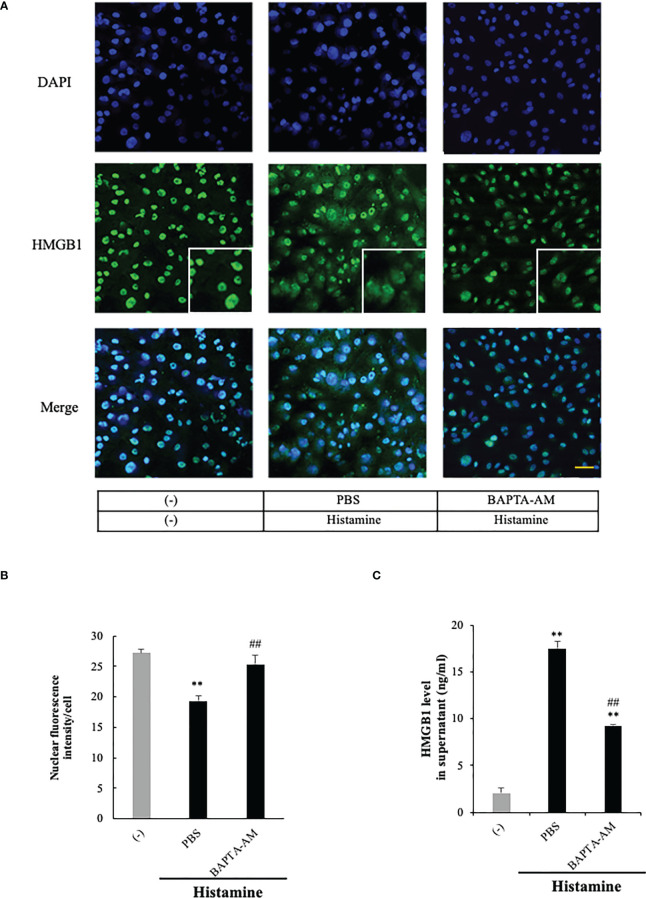
Histamine-induced HMGB1 release occurred in a Ca^2+^- dependent manner. **(A)** Confluent EA.hy 926 cells were preincubated with the Ca^2+^ chelator BAPTA-AM (5 uM) or PBS for 1 h. The cells were then stimulated with histamine (1 μM) for 12 h. HMGB1 translocation in VECs was observed by immunostaining. Scale bar = 10 μm. **(B)** The results were quantified by ImageJ software and are expressed as the ratio of total nuclear HMGB1 intensity/cell numbers. **(C)** The release of HMGB1 into the cell culture medium was measured by ELISA. The results shown are the means ± SEM of three experiments, (n=3 per group). One-way ANOVA followed by the *post hoc* Fisher test. ^**^p<0.01 vs. control in the absence of histamine, ^##^p<0.01 vs. histamine-PBS group.

The intracellular signaling systems mediated by H_1_R have been well documented (14, 15). H_1_R stimulation activates phospholipase Cβ *via* G_q/11_ and the resultant production of IP3 in turn induces calcium mobilization from ER calcium stores. Therefore, if the event of HMGB1 mobilization induced by H_1_R stimulation occurs downstream of calcium mobilization, the blocking of calcium signals may lead to the diminution of HMGB1 mobilization. **Figure 4** shows that a membrane-permeable calcium chelator, BAPTA-AM (5 μM), significantly inhibited the mobilization as well as the release of HMGB1 induced by histamine (1 μM) (**Figures 4A–C**), suggesting that free calcium in the cytosolic compartment plays a fundamental role in the mobilization of HMGB1.

### Effects of adrenaline and noradrenaline on histamine-induced translocation of HMGB1 in VECs

**Figure 5:**
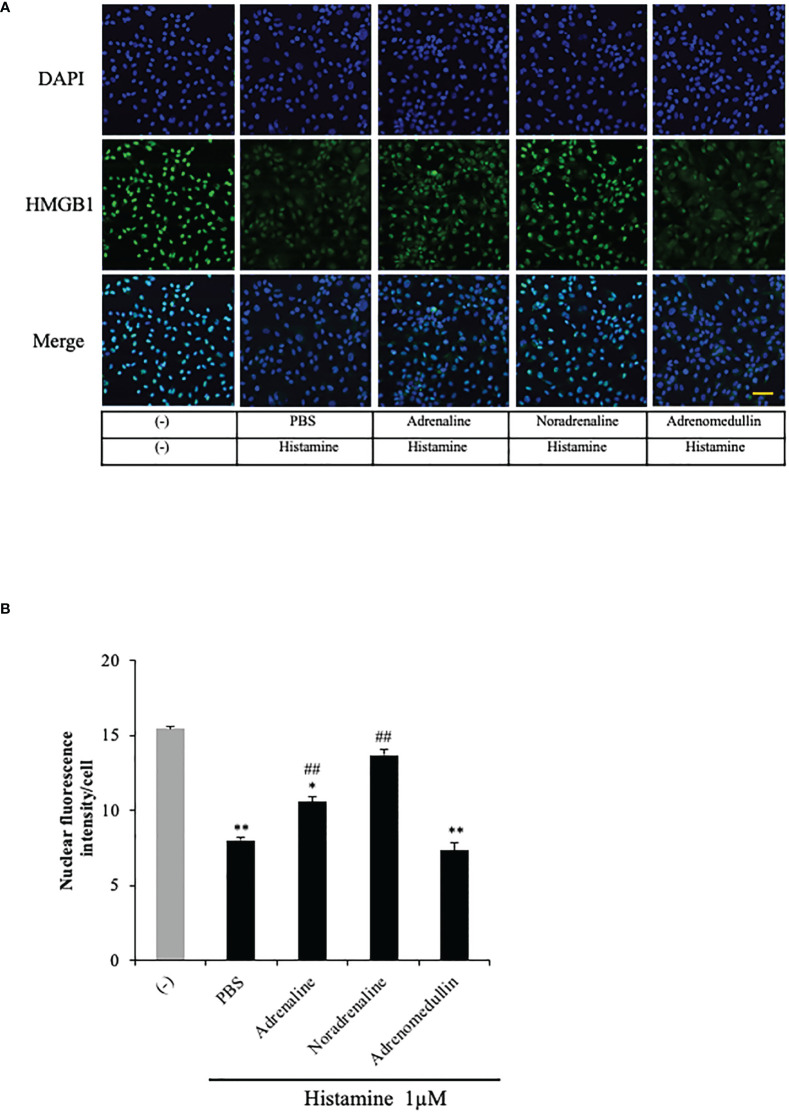
Effects of adrenaline and noradrenaline on histamine-induced HMGB1 translocation in VECs. **(A)** EA.hy 926 cells were preincubated with adrenaline, noradrenaline or adrenomedullin (5 μM) for 1 h before stimulation with histamine (1 μM). The translocation of HMGB1 was determined by immunostaining. The results are representative of ≥5 experiments. Scale bar = 10 μm. **(B)** The HMGB1 translocations were quantified by ImageJ software and expressed as the ratio of nuclear HMGB1 intensity against the total cell number. The results shown are the means ± SEM of five different experiments, (n=5 per group). One-way ANOVA followed by the *post hoc* Fisher test. ^*^p<0.05, ^**^p<0.01 vs. control in the absence of histamine, ^##^p<0.01 vs. histamine-PBS group.

Because HMGB1 has been reported to induce endothelial contraction and hyperpermeability

(25, 26), the results obtained above imply a novel mechanism of histamine-induced anaphylactic shock—namely, histamine could cause the HMGB1 release from VECs through H_1_R and lead to the hypotension. In an anaphylactic emergency, adrenaline administration is the first-choice treatment for restoring the blood pressure. Accordingly, we examined the effects of adrenaline and noradrenaline on the histamine-induced HMGB1 in endothelial cells. The results showed that both adrenaline (5 μM) and noradrenaline (5 μM) effectively inhibited the HMGB1 translocation (**Figures 5A, B**). Adrenomedullin is a potent long-acting vasodilatory peptide which contains 52 amino acids and is produced in vascular endothelial cells. Although adrenomedullin has anti-inflammatory activity, however, adrenomedullin (5 μM) did not show any effects on histamine-induced translocation of HMGB1.

### Effects of anti-HMGB1 mAb on compound 48/80-induced anaphylactic shock in rats

**Figure 6:**
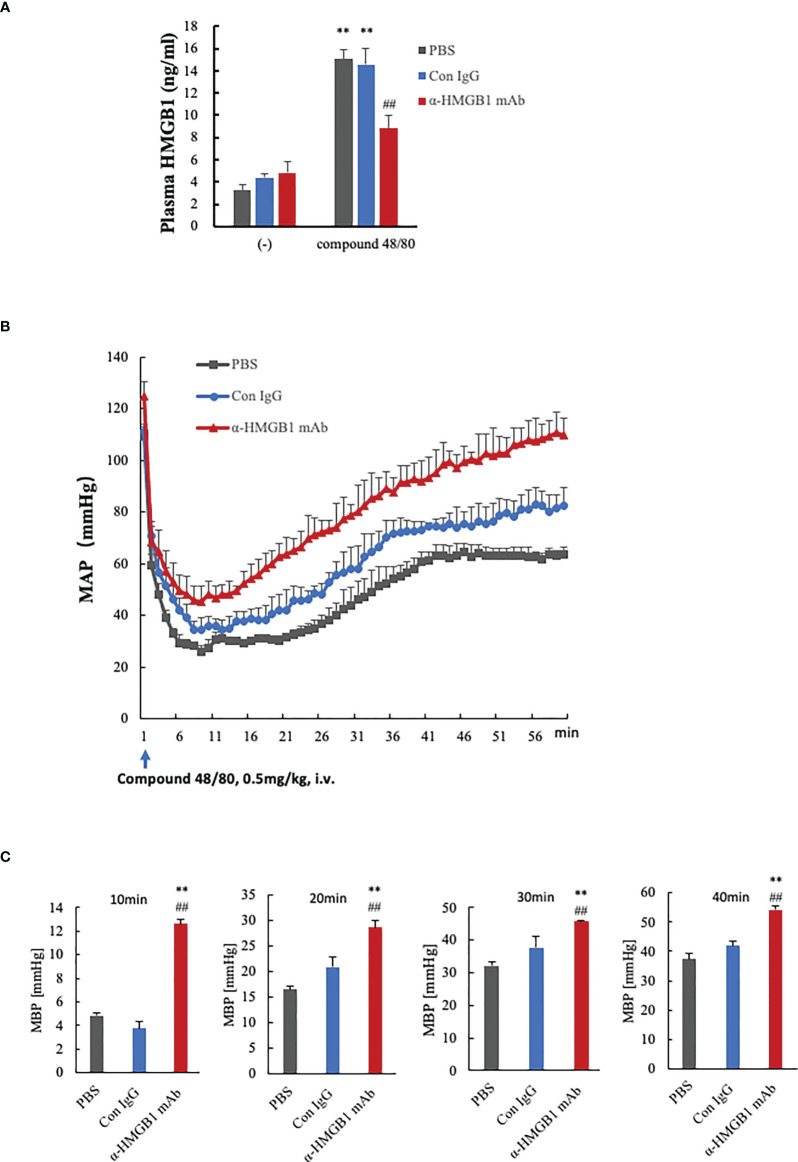
Effects of anti-HMGB1 mAb on a compound 48/80-induced rat model of anaphylactic shock. Wistar Rats were given compound 48/80, a mast cell degranulation agent, at a dose of 0.5 mg/kg through the tail vein to induce anaphylactic shock. One minute after compound 48/80 injection, the rats were treated with PBS, anti-KLH mAb (2 mg/kg) or anti-HMGB1 mAb (2 mg/kg) through the tail vein. Measurement of hemodynamic parameters was performed every 5 min for a period of 30 min before the compound 48/80 challenge. The hemodynamic parameters were recorded for 60 min at 1 min intervals after the compound 48/80 challenge. **(A)** Plasma levels of HMGB1 in rats at 10 min after compound 48/80 injection were determined by ELISA. **(B)** Records of mean arterial blood pressure (MAP, mmHg) of rats treated with PBS, control mAb or anti-HMGB1Ab. MAP was recorded every minute until the end of the experiment at 60 min. Each point represents the means ± SEM of six rats, (n=6 per group). All results are the mean ± SEM of five different experiments. One-way ANOVA followed by the *post hoc* Fisher test. ^**^p<0.05 vs. PBS, ^##^p<0.05 vs. Control IgG. **(C)** The absolute increase in the value of MBP from the lowest after AS induction to the indicted time was quantified in each group. one-way ANOVA followed by the *post hoc* Fisher test. **p<0.05 vs. control, ^##^p<0.05 vs. Con IgG.

Histamine is a major inducer of vascular hyperpermeability, and is thus a central component of permeability-related human pathologies, such as allergy and anaphylaxis. Histamine in the granules of mast cells and basophils is released from preformed stores in an antigen-IgE-dependent manner, leading to a rapid increase in vascular permeability within minutes and causing hypotension. We established an anaphylactic shock model in rats by the intravenous injection of compound 48/80, a mast cell degranulator. We first collected blood samples from rats 10 min after injection with compound 48/80, and found that plasma HMGB1 levels were significantly increased in the compound 48/80-treated rats compared with the non-treated controls (**Figure 6A**). The post-treatment of rats with the anti-HMGB1 mAb (2 mg/kg, i.v.) reduced the increase in plasma HMGB1 levels compared with the control IgG- and PBS-treated groups (**Figure 6A**). Then, we measured the mean arterial blood pressure of rats and observed a sharp drop of blood pressure from 120 to 30 mmHg at 10 min after the injection of compound 48/80. This hypotensive state in PBS-treated rats continued until 20 min post-injection and then gradually recovered to the level of 60 mmHg by 60 min post-injection (**Figure 6B**). The post-treatment of rats with the anti-HMGB1 mAb reduced the maximal decreased level of mean arterial blood pressure and accelerated the recovery of hypotension significantly. At the end of the recording period (at 60 min), the mean arterial blood pressure in the anti-HMGB1 mAb-treated group was above 100 mmHg (**Figure 6B**). **Figure 6C** shows the magnitudes of recovery of the mean arterial blood pressure at the indicated time points from the lowest blood pressure. These results demonstrated that the compound 48/80-histamine-induced rapid hypotension was at least partly caused by the HMGB1 release, and that the neutralization of circulating HMGB1 by anti-HMGB1 mAb inhibited the anaphylactic hypotension.

## Discussion

The results of the present study clearly demonstrated that histamine induced the mobilization of HMGB1 from nuclei to the extracellular space through the cytosolic compartment in VECs (**Figure 1**). The release of HMGB1 induced by histamine was concentration- and time-dependent. The translocation of HMGB1 appeared to proceed in a manner quite similar to those induced by LPS and TNF-α (27). The experiments using receptor subtype-specific agonists showed that 2-pyridyl ethylamine was a specific agonist for H_1_-receptors, and produced a similar HMGB1 mobilizing activity to histamine, whereas H_2_-receptor agonist (4-methylhistamine) (30) did not (**Figures 3A, B**). Also, *d*-chlorpheniramine, a specific antagonist for H_1_-receptor, concentration-dependently inhibited the HMGB1 translocation induced by histamine, while an H_2_-receptor antagonist (famotidine) or H_3/4_ antagonist (thioperamide) (31) did not produce any effects (**Figures 2B–D**). Taken together, these results indicated that the only receptor subtype involved in the action of histamine on HMGB1 mobilization in VECs was the H_1_-receptor.

It is well known that H_1_ receptor stimulation by histamine causes remarkable functional changes in VECs, including NO production, eNOS induction, upregulation of surface expression of E-selectin, IL-8 secretion and cell contraction (32). The expression of E-selectin induces the rolling of leukocytes on the endothelial cells through the interaction with PSGL-1 (33), which facilitates the inflammatory responses (34). Moreover, NO produced in the endothelial cells diffuses into the smooth muscle cells, leading to the dilatation of vessels (35). The contraction of endothelial cells of postcapillary venules leads to the leakage of plasma proteins and the formation of tissue edema. A previously reported *in vivo* experiment showed that histamine-induced hyperpermeability was dependent predominantly on NO-mediated dilation of vascular smooth muscle and the subsequent blood flow increase, and partially on PKC/ROCK/NO-dependent endothelial barrier disruption (36). HMGB1 can be actively released from the VECs upon exposure to various stimuli, such as LPS or TNF-α (29). The released HMGB1 can further activate endothelial cells, leading to up-regulation of the cell adhesion molecules ICAM-1, VCAM-1, and E-selectin, and is involved in the cytokine secretion in cells (37, 38). The released HMGB1 has also been found to induce early EC barrier disruption, with a potential molecular mechanism being activation of the RhoA/ROCK1 signaling pathway by HMGB1 *via* RAGE (39). These similarities between HMGB1 and histamine in the regulation of cell inflammatory and endothelial cell permeability indicate a possible relationship between them in the vascular system.

Anaphylaxis is triggered by a specific antigen binding to IgE antibody on the surface of mast cells and basophils (40). Histamine is a biogenic amine stored in the granules of mast cells and basophils and a well-known mediator of anaphylaxis (41). The massive release of granule constituents from these cells causes a rapid decrease in arterial blood pressure (42). In the present study, we mimicked the anaphylactic response by intravenous injection of compound 48/80, a mast cell stimulator, in rats. The compound 48/80-induced hypotension was accompanied by an elevation of plasma HMGB1 (**Figure 6A**). The post-treatment of rats with a neutralizing antibody against HMGB1 significantly accelerated the recovery from the hypotensive response induced by compound 48/80 (**Figure 6B**). These results strongly suggest that HMGB1 is involved in the hypotensive response to compound 48/80. They also suggest the possibility that histamine released from mast cells in response to compound 48/80 induced the translocation and extracellular release of HMGB1 from VECs.

HMGB1 is expressed ubiquitously in almost all kinds of cells, while not all kinds of cells can actively release HMGB1 after stimulation. Although there is little information about the HMGB1 release from mast cells, one report showed the lack of release of HMGB1 from the murine mast cell line C57 and the human mast cell line HMC-1.2 after stimulation with different cytokines and antigen-IgE (43). In our study, we observed that HMGB1 can be released from vascular endothelial cells after stimulation with histamine *in vitro*. During the anaphylactic response, histamine released from mast cells and basophils gets into blood stream and can easily access to the vascular endothelial cells, therefore, we speculated that the HMGB1 was mainly released from vascular endothelial cells in this process although the release of HMGB1 from other types of cells could not be excluded.

Piao et al. reported that recombinant HMGB1 alone induced a release of β-hexosaminidase associated with the up-regulation of TLR4, Myd88 and NF-kB nuclear translocation in rat basophil leukemic cell line, RBL-2H3, whereas the knockdown of HMGB1 in RBL-2H3 by siRNA of HMGB1 suppressed the expression of TLR4/Myd88-signaling molecules and reduced the secretory response induced by antigen-IgE (44). These results suggested that endogenous HMGB1 may be involved in activation of signaling machinery in basophils and play an important role in the secretory response of basophils. On the other hand, there is little information about the involvement of endogenous HMGB1 in mast activation and secretion. Further works are necessary on this line. Collectively, the results of this study suggest that HMGB1 released from VECs into the blood stream by histamine is at least partly involved in the hypotensive response to compound 48/80 in a paracrine manner. It is noteworthy that the agents currently used for clinical treatment of anaphylaxis, adrenaline and noradrenaline, efficiently inhibited the nuclear translocation of HMGB1 induced by histamine in the present study (**Figures 5A, B**). Consequently, it is likely that the clinical therapeutic effects of these catecholamines may be ascribed at least in part to the inhibition of HMGB1 release from VECs and the subsequent protection of endothelial cells from the effects of HMGB1.

Zhang et al. (25) observed a direct action of recombinant HMGB1 on a reconstituted blood-brain barrier composed of brain VECs, pericytes and astrocytes. In this system, HMGB1 induced a contractile response in both endothelial cells and pericytes, leading to an increase in BBB permeability. The HMGB1 release from neurons was evident after the brain ischemia/reperfusion or brain trauma in rats (24, 25), indicating that HMGB1 was increased in both plasma and the CNS. The released HMGB1 probably reached the BBB and impaired its structure and function, leading to the brain edema formation and associated brain injury. The treatment with anti-HMGB1 neutralizing antibody used in the present study efficiently inhibited the BBB disruption and the accompanying inflammatory responses that were mediated by cytokine and inflammation-related molecules in the brain (24, 25). Accordingly, anti-HMGB1 mAb therapy may be very useful to prevent the actions of HMGB1 on VECs. In the case of impairment of BBB integrity in the brain, it has been suggested that both RAGE and TLR4 are involved in the direct effects of HMGB1 on endothelial cells and pericytes (25, 26). Therefore, it might be possible that the HMGB1 released by histamine in turn stimulates endothelial cells in an autocrine and a paracrine fashion. There are several kinds of important factors that induce the contraction of endothelial cells and increase capillary permeability, such as bradykinin, leukotriene C4 and PAF (45–47). However, it remains to be determined whether all of these factors can induce translocation and release of HMGB1 from endothelial cells.

The effects of histamine were thought to be mediated by a rapid and transient increase in cytosolic calcium levels *via* the production of IP3 by activation of phospholipase-Cβ. We also observed that the HMGB1 translocation induced by histamine was Ca^2+^ dependent (**Figures 4A–C**). The initial intracellular signaling triggered by H_1_-receptor stimulation may induce the rapid production of NO, leading to a quick vasodilatory response through diffusion into the smooth muscle cells. However, the mobilization and release of HMGB1 was time-dependent over hours as in the case of eNOS induction, the secretion of IL-8 and von Willebrand factor, and the surface expression of E-selectin. Thus, H_1_-receptor stimulation appears to induce rather long-lasting cellular effects by the downstream signaling events, leading to the individual cellular responses. The H_1_-receptor upregulation observed in the present study may be one such long-lasting response, which would be consistent with the results reported previously (48).

The intracellular signals triggered by H_1_ receptor stimulation include Gq/G_12_ activation, phospholipase C activation and IP3-induced calcium mobilization (49–51). Therefore, it is quite probable that the HMGB1 mobilization occurs downstream of these events. Previously, we observed that an HMGB1 translocation induced by TNF-α or LPS was similar to that induced by histamine in present study. Since the intracellular signalings induced by TNFR1/2 and TLR4/MD2/CD14 are quite different from that induced by histamine H_1_ receptor, a G protein-coupled receptor. At present, little is known about the mechanism of HMGB1 mobilization, except for the possible chemical modification of HMGB1 (52). Therefore, the pathway leading to the HMGB1 translocation and release in VECs by histamine stimulation need to be studied in the future.

## Data availability statement

The original contributions presented in the study are included in the article/**Supplementary Material**. Further inquiries can be directed to the corresponding author.

## Ethics statement

The animal study was reviewed and approved by Okayama University.

## Author contributions

SG conceived the study, designed the experiments, analyzed data, and wrote the manuscript. MN and HW for editing the manuscript. KL, WK, and DW performed the animal experiments. KT and HQ for critically reviewing the manuscript. All authors contributed to the article and approved the submitted version.

## Funding

This work was supported by funds from the National Key R&D Program of China (Grant No. 2021YFE0109300). The work was also supported by a MHLW research on chronic pain Program Grant (JPMHLW22FG1003), a Grant-in-Aid for Scientific Research (no.19H03408 to MN), a Grant-in-Aid for Young Scientists (no. 17K15580 to HW) from the Japan Society for the Promotion of Science (JSPS). The author S.G. was supported by a funding from Tsinghua University-Peking University Joint Center for Life Sciences.

## Conflict of interest

The authors declare that the research was conducted in the absence of any commercial or financial relationships that could be construed as a potential conflict of interest.

## Publisher’s note

All claims expressed in this article are solely those of the authors and do not necessarily represent those of their affiliated organizations, or those of the publisher, the editors and the reviewers. Any product that may be evaluated in this article, or claim that may be made by its manufacturer, is not guaranteed or endorsed by the publisher.
