# Supplementary material for: Histamine induced high mobility group box-1 release from vascular endothelial cells through H1 receptor
Source: Front Immunol. 2022 Oct 5;13:930683. doi: 10.3389/fimmu.2022.930683 (PMC9583674; doi:10.3389/fimmu.2022.930683)
Supplement: Supplementary file 1 [file DataSheet_1.docx]

**Figure S1. Cell viability of EA.hy 926 cells after treatment with antagonists or agonists of histamine receptors.** EA.hy 926 cells were pre-incubated with 1 μM *d*-chlorpheniramine (H1-selective antagonist), famotidine (H2-selective antagonist) or thioperamide (H3/H4-selective antagonist), adrenaline, noradrenaline or adrenomedullin (5 μM) before stimulation with histamine (1 μM) for 12 h. EA. hy 926 cells treated with 2-pyridylethylamine (H1-selective agonist) or 4-methylhistamine (H2-selective agonist) for 8 h. The cells were then incubated with MTT at 37℃ for 4 h by adding 10 μl of 5 ng/ml MTT solution into each well. After the removal of the cell supernatant, 200 μl of DMSO was added into each well to dissolve the crystals. The OD value was recorded using microplate reader at 570 nm wavelength. All results are the means ± SEM of three different experiments, n=5.

**

**

**Figure S2. HMGB1 mRNA expression in EA.hy 926 cells after treatment with antagonists or agonists of histamine receptors.** EA.hy 926 cells were cultured with antagonists (*d*-chlorpheniramine, famotidine, thioperamide) or agonists (2-pyridylethylamine or 4-methylhistamine) of histamine receptors. The expression of HMGB1 at the mRNA level was measured by quantitative RT-PCR. The results were normalized to the expression of β-actin and are expressed as the means ± SEM of five determinations. One-way ANOVA followed by the post hoc Fisher test. **p<0.01 vs. control in the absence of any agonist.

**Supplementary Table**

RT-PCR Primer Sequence

| mRNA Sense primer Anti-sense primer |
| --- |
| H_1_R 5'-CATTCTGGGGGCCTGGTTTCTCT-3' 5'-CTTGGGGGTTTGGGATGGTGACT-3' |
| H_2_R 5'-CCCGGCTCCGCAAC CTGA-3' 5'-CTGATCCCGGGCGACCTTGA-3' |
| H_3_R 5'-CTTCCTGCCCT A GCAGTT-3' 5'-GCAGAGAACAGCTTCGAGG TT-3' |
| HMGB1: 5'-AGATATGGCAAAAGCGGACAAG-3' 5'-TCAGAGCAGAAGAGAAGAAGG-3' |
| β-actin: 5'-AGCGGGAAATCGTGCGTG-3' 5'-CAGGGTACATGGTGGTGCC-3' |
